# Validation and application of health utilities index in Chinese subjects with down syndrome

**DOI:** 10.1186/s12955-014-0144-x

**Published:** 2014-10-14

**Authors:** Winnie Ka Yan Mok, Wilfred Hing-Sang Wong, Gary Tsz Kin Mok, Yoyo Wing Yiu Chu, Frederick Ka Wing Ho, Chun Bong Chow, Patrick Ip, Brian Hon-Yin Chung

**Affiliations:** Department of Paediatrics and Adolescent Medicine, Queen Mary Hospital, Li Ka Shing Faculty of Medicine, The University of Hong Kong, Hong Kong Special Administrative Region, People’s Republic of China

**Keywords:** Down syndrome, Health-related Quality of Life, Health Utility Index, Chinese, Chronic health conditions

## Abstract

**Objectives:**

The objectives of the study were (1) to validate the Chinese version of Health Utilities Index (HUI-Ch); (2) to examine the Health-related Quality of Life (HRQoL) of Chinese subjects with Down syndrome (DS); and (3) to study the impact of chronic health conditions on HRQoL of Chinese with DS.

**Methods:**

The multiple choice questionnaire for scoring Health Utilities Index Mark 2 (HUI2) and Health Utilities Index Mark 3 (HUI3) was translated and validated. In addition to the HRQoL scores from HUI2 and HUI3, proxy-data on socio-demographics, and 10 common chronic health conditions for people with DS were collected and analyzed. Data analysis involves multiple imputation and multiple regression analysis to predict variations in HRQoL in relation to different factors. Lastly, a gradient interval was constructed on the number of chronic health conditions in relation to HRQoL.

**Results:**

HUI-Ch was validated according to standard guidelines. People with DS were found to have a lower HRQoL as compared to the general population, with the majority categorized as moderate or severe on the scale. Behavioral and hearing problems on HUI2, and hearing problems on HUI3 were found to be statistically significant predictors of a lower HRQoL score. A significant gradient relationship existed showing when the number of health problems increased, the HRQoL scores decreased.

**Conclusions:**

HUI-Ch is a valid instrument to assess HRQoL. It can have broad application in Chinese subjects with DS including the study of the impact of different chronic health conditions on their quality of life. The quantifiable nature of HUI-Ch will facilitate longitudinal study on the well-being of subjects with DS and evaluation of effectiveness of intervention programs in the near future.

**Electronic supplementary material:**

The online version of this article (doi:10.1186/s12955-014-0144-x) contains supplementary material, which is available to authorized users.

## Objectives

Down syndrome (DS), also known as trisomy 21, is the most common chromosome abnormality characterized by having an extra copy of chromosome 21. Globally, the incidence ranges from 1 in 650 to 1000, estimating the total number of people with DS to be 6 million [1,2]. In Hong Kong, a similar incidence rate of 1 in 767 live births was found [3].

Epidemiologic studies have identified people with DS to have an increased risk of chronic health conditions including cardiac, endocrine, gastrointestinal, immunological, respiratory, musculoskeletal, as well as oncological diseases [4-10]. It has been suggested that people with DS has a unique aging profile, with premature onset and rapid progressing manifestation of age-related conditions such as menopause, sensory impairments, thyroid dysfunction, diabetes, sleep apnea, and Alzheimer’s disease [11-20]. Their functional capability is limited due to mental impairment, affecting their learning abilities, verbal working memory, and receptive language [21,22]. Behavior problems such as impaired social development, impulsive behavior, poor judgment, and short attention span are also commonly observed amongst people with DS [1].

Health-related Quality of Life (HRQoL) is a multi-dimensional concept reflecting life quality related to physical, mental, emotional and social functioning. As defined by the World Health Organization (WHO), the HRQoL comprises of six domains: physical health, psychological well-being, level of independence, social relationships, environment, and personal beliefs. In subjects with DS, the diverse set of physical and development impairments is likely to adversely impact their HRQoL. As demonstrated by a study using the TNO-AZL Children’s Quality of Life questionnaire, a significant deficit in HRQoL was observed in a group of 8-year-old children with DS as compared to a normative population [22]. Another study found that recurrent respiratory tract infections had a large adverse impact on the HRQoL of children with DS [23].

As the longevity of people with DS increased over the years, the life expectancy was observed to be around 60 years of age [10,13,24-26]. Published studies evaluating HRQoL of people with DS were so far limited and primarily focused on children, assessing only one disease group, and of western population. The HRQoL of adults with DS have not been well studied, and outcome measures may also be different in Chinese. Therefore, the objectives of this study were: 1) to validate the Chinese version of Health Utilities Index (HUI-Ch), 2) to determine the HRQoL of both adult and paediatric Chinese subjects with DS, and 3) to study the relationship between chronic health conditions and HRQoL of Chinese subjects with DS.

## Methods

Two health-state classification systems, Health Utilities Index Mark 2 (HUI2) and Health Utilities Index Mark 3 (HUI3) were used to measure HRQoL. These instruments are non-disease specific index applicable to individuals over the age of 5 and have been shown to be responsive to various chronic diseases such as rheumatoid arthritis [27], type 2 diabetes [28], and stroke [29]. HUI2 and HUI3 are 2 independent yet complementary systems measuring HRQoL. Each of HUI2 and HUI3 includes a generic comprehensive health status classification system and a generic HRQoL utility scoring system. Outlines of the different attributes measured between the two HUI system were included in the Additional files.

The 15-multiple choice questionnaire allows scoring subjects according to HUI2 and HUI3. HUI2 describes an individual’s functional health status using 7 health attributes (Sensation, Mobility, Emotion, Cognition, Self-Care, Pain and Fertility) distinguished by 3–5 descriptive levels (see Additional file 1: Table S1) while HUI3 describes using 8 health single-attributes (Vision, Hearing, Speech, Ambulation, Dexterity, Emotion, Cognition and Pain) distinguished by 5–6 descriptive levels (see Additional file 2: Table S2). A multiplicative scoring algorithm using the individual health attributes of HUI2 and HUI3 yield the multi-attribute utility scores, which is the numerical measurement for HRQoL. HRQoL score has an interval-scale property with score of 0.00 being conventional dead to 1.00 being in perfect health [30-35].

### Translation and validation of HUI (traditional Chinese version)

English-language HUI questionnaire obtained from Health Utilities Inc. (HUInc) was translated into traditional Chinese language (HUI-Ch) for adaptation and use in Chinese population. The research team followed the stringent translation and adaptation procedures of HUI instrument in accordance to the guideline set out by HUInc. This included forward translation by an academic translator, followed by 2 backward translations by a board-certified genetic counselor and a research assistant independently. Discrepancies were identified, analyzed, and revised internally and reviewed by HUInc personnel as well as experts in the field to ensure linguistic equivalence between the original and translated version. Acceptability testing on 5 healthy Chinese adults was also conducted to ensure consistency between English and Chinese version of HUI.

Chinese subjects with DS were recruited in collaboration with the Hong Kong Down Syndrome Association (HKDSA). Staff of HKDSA invited parents or caregivers of people with DS to complete a questionnaire and a proxy version of the Chinese translated version of HUI at various activities hosted by HKDSA. Detailed explanations were provided to participants and their families before they signed the consent. Verbal instructions were provided to respondents on how to fill in the questionnaire and on the clarification of any unclear items. The study aimed at recruiting as many subjects as possible and the recruitment was carried out over the 6 months period from Dec 2012 to May 2013. The participants did not receive any incentives and none of them requested to leave the study. Because of varying intellectual ability of the people with DS, respondents were the parents or caregivers, whom provided answers in lieu of the individual with DS. An information sheet was provided to each participant and his/her family to clearly inform about the purpose and details of the survey and provide participant a contact phone number to reach our research team. Consent form was signed by each participant’s family member before he/she joined the study. To protect participant’s privacy, a unique study number (instead of personal ID number) was assigned to each participant and the data were all kept in a computer with protected password locked in our University office.

### Measures

Data on the HRQoL, DS subject’s demographics, and chronic health conditions (as defined by local population-based health survey [36]) were collected and used for analysis. Ten chronic health conditions more prevalent in DS [5,8,22,37-39] were selected and categorized into 2 groups, namely physical health problem and developmental-behavioral problem. The five physical health problems included cardiac diseases, endocrine problems, growth problem, sleep disturbances, and chronic orthopedic bone/joint problems. The five developmental-behavioral problems included behavior problems, speech problems, learning problems, hearing impairment/deafness, and vision problems. Respondents were asked to report if the health condition has been observed in the individual with DS.

### Data analysis

HUI data were analyzed according to the manual provided by HUInc. The tabulated HRQoL scores were then categorized into 4 disability levels (none, mild, moderate, severe) as proposed by the HUI developers. Such categorization method has been validated as appropriate for assessing disability levels for comparison to general population [40]. The disability level descriptions are summarized in Additional file 3: Table S3. Missing data were treated using multiple imputations with 5 times imputations. Unpaired t-tests were used to test the univariate relationship between HRQoL scores and demographics and different chronic health conditions. Significant factors correlating to HRQoL scores were then put into multiple linear regression models. R statistical software version 3.0.1 was used for the analysis. A p-value with 2-tail < 0.05 was treated as significant. Exclusion criteria included non-Chinese and those individuals with DS under the age of 5.

This study received ethics approval from the Institutional Review Board of the University of Hong Kong/Hospital Authority Hong Kong West Cluster.

## Results

### Characteristics of study population

A total of 109 individuals with DS participated in this study. As summarized in Table 1, 79 (72%) were adults while 30 (28%) were aged 18 or below. Sixty-one (56%) of participants were male. All were Chinese descendants; with 99% of them being Hong Kong permanent residents using Cantonese as their primary language. As the survey was proxy-assessed, we found that 89% of the respondents were mothers of the subject with DS, 6% were fathers, 4% were siblings, and 2% were other relatives.

**Table 1:** **Characteristics of subjects with Down syndrome**

	**n**	**%**
Age (in years)		
5-18	30	28%
>18	79	72%
Gender		
Male	61	56%
Female	48	44%
Race		
Chinese	109	100%
Hong Kong Resident Status:		
Permanent resident	108	99%
Singly- non-permanent resident	0	0%
Doubly- non-permanent resident	1	1%
Non-residence	0	0%
Primary Language spoken:		
Cantonese	108	99%
Putonghua	1	1%
Proxy-relation:		
Mother	97	89%
Father	6	6%
Sibling	4	4%
Others	2	2%

### Self-reported chronic health conditions

The self-reported chronic health conditions were categorized into physical health and developmental-behavioral problems. Physical health problems included 10 (9%) cardiac disease, 24 (22%) endocrine problems, 27 (25%) growth problems, 18 (17%) sleep disturbance issues, and 11 (10%) chronic orthopedic bone or joint problems. Developmental-behavioral problems included 18 (17%) behavioral problems, 58 (53%) speech problems, 55 (50%) learning problems, 21 (19%) hearing impairment or deafness, and 50 (46%) vision problems.

### HRQoL analysis

**Figure 1:**
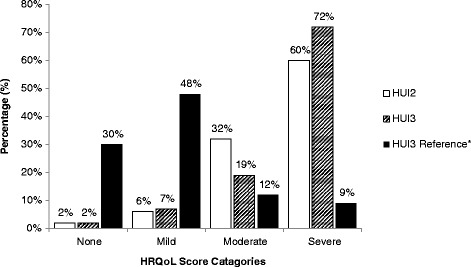
**HUI2 and HUI3 HRQoL scores in individuals with DS (n = 109).** *HUI3 Reference Source: 2005 Canadian Community Health Survey, which represents the HRQoL of 18,271 Canadians with no chronic conditions at all [41].

Categorization of HRQoL scores into disability level shown in Figure 1 illustrated that the majority of Chinese people with DS were in the ‘severe’ disability group. As shown in Table 2, analysis using unpaired *t*-test and multiple regression for both HUI2 and HUI3 showed that demographic factors (gender and age) and physical conditions did not affect their HUI2 and HUI3 HRQoL scores.

**Table 2:** **Chronic health conditions in HUI2**/**HUI3 scores**

	**HUI2**			**HUI3**		
	**Two-sample** ***t*** **-test**		**Multiple regression**	**Two-sample** ***t*** **-test**		**Multiple regression**
	**Mean (SD)**	**p**	**p**	**Mean (SD)**	**p**	**p**
**Physical conditions**						
Endocrine problems		0.17	-		0.02*	0.20
No	0.74 (0.15)			0.59 (0.26)		
Yes	0.69 (0.15)			0.42 (0.28)		
**Developmental**-**behavioural conditions**						
Behavioural problems		<0.01*	0.04*		0.02*	0.28
No	0.75 (0.14)			0.58 (0.27)		
Yes	0.64 (0.16)			0.42 (0.26)		
Speech problems		0.01*	0.47		<0.01*	0.50
No	0.77 (0.15)			0.65 (0.26)		
Yes	0.69 (0.14)			0.47 (0.26)		
Learning problems		0.04*	0.94		0.01*	0.54
No	0.76 (0.17)			0.63 (0.26)		
Yes	0.71 (0.13)			0.48 (0.27)		
Hearing problems		<0.01*	0.03*		<0.01*	0.03*
No	0.75 (0.14)			0.59 (0.25)		
Yes	0.64 (0.16)			0.39 (0.28)		
Vision problems		0.03*	0.44		<0.01*	0.35
No	0.76 (0.14)			0.62 (0.24)		
Yes	0.70 (0.16)			0.47 (0.29)		

Base on the death to perfect health scale (0.00 – 1.00).

*p-value < 0.05.

Evaluating the impact of different factors on HUI2 HRQoL scores indicated that those having developmental-behavioral conditions (problems in behavioral (p < 0.01), speech (p = 0.01), learning (p = 0.04), hearing (p < 0.01), and vision (p = 0.03)) had a significantly lower HUI2 HRQoL score. Among them, only behavior problems (p = 0.04) and hearing problems (p = 0.03) were found to be significant indicators for a lower HRQoL score by multiple regression. We found lower HUI3 scores among those who had all 5 developmental-behavioral conditions (problems in behavioral (p = 0.02), speech (p < 0.01), learning (p = 0.01), hearing (p < 0.01), and vision (p < 0.01)) and among those who had at least one physical conditions (endocrine problems (p = 0.02)). Hearing problem (p = 0.03) was the only significant factor accounted for a lowered HRQoL score by multiple regression.

**Figure 2:**
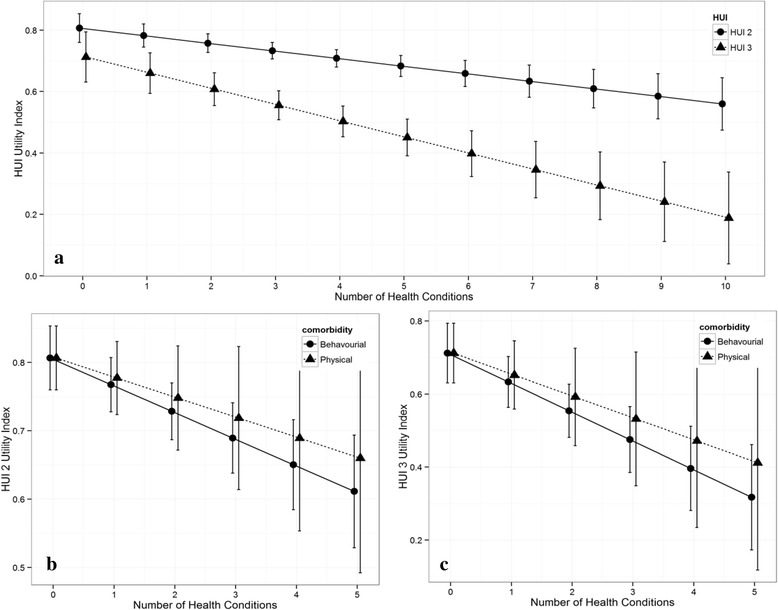
**Relationship between health-related quality of life and chronic health conditions.** **(a)** Association between HRQoL score from HUI2 and HUI3 and number of any health conditions. The HRQoL scores of both HUI2 and HUI3 were both significant as the number of health conditions increase since the p-values for both HUI2 and HUI3 are less than 0.001. However only the HRQoL score of HUI3 was significantly different in the presence of co-morbidities, p-value less than 0.05. **(b)** and **(c)** shows the association between HRQoL scores from HUI2 and HUI3 and the number of physical or developmental-behavioral condition respectively. The HRQoL score of HUI2 and HUI3 were significantly different as the number of developmental-behavioural conditions increase since there p-values were both less than 0.001.

Figure 2 showed HUI2 and HUI3 in relation to increasing number of chronic health conditions the individuals with DS experienced. Both HUI2 and HUI3 classification system yielded a statistically significant negative relationship where HRQoL scores decreased as the number of health problems increased. The extent of decrease detected is more sensitive in HUI3 than HUI2. Assessments of HUI2 and HUI3 in relation to increasing number of any of the 5 physical health problems or any of the 5 developmental-behavioral problems individually are represented in Figure 2b) and c). Worth to note, a greater inverse dosage effect was observed on HRQoL deficit as the number of developmental-behavioral conditions increased as compared to physical health problems. The negative linear relationship between number of chronic conditions and HRQoL was found to be significant (p < 0.001) for developmental-behavioural conditions but not for physical health problems (p > 0.05) in both HUI2 and HUI3 classification method.

## Discussion

The goal of this study was to develop and validate the use of HUI-Ch in the Chinese population and to investigate the HRQoL of people with DS in Hong Kong. We have described the process for translation of the HUI2 and HUI3 questionnaire into traditional Chinese and the validation process. HUInc has validated and approved more than 35 other languages so far, including languages such as German [42], Spanish [43] and French [44]. The standard translation process recommended by HUInc is a published guideline by Guillemin et al. on translation and cultural adaptation of HRQoL questionnaires [45]. Validation process was completed with direct oversight by HUI developers, and the HUI-Ch received final approval from the developers of HUI.

The HUI instrument is a generic preference-scored system for measuring health status and HRQoL for a broad range of subjects. The sample of our study covers a broad age range, assessing the HRQoL of 109 people with DS from age of 5 and above. As compared to findings of local population health survey [36], we found a much higher occurrence rates of physical health and developmental-behavioral problems amongst DS subjects in this study, signifying that the chronic health conditions were more prevalent in DS. This study also demonstrated that the HRQoL of people with DS is less favorable than a reference group with no disability from a general population cohort without any reports of chronic illness [41]. When comparing to the HRQoL of different conditions found in past studies that used HUI, the HRQoL of subjects with DS in the current study is found to be generally lower. This can be seen in Table 3. Since the current study is the first HRQoL study using HUI both in Chinese and in people with DS, this makes it difficult to make direct comparison with other relevant studies and we were only able to compare with HRQoL of other patient groups in other countries. After publication, we will conduct further studies in local Chinese population in Hong Kong and this will allow us to compare findings among populations with different medical conditions and further confirm the validity of HUI-Ch.

**Table 3:** **Comparison of HUI3 scores in studies of different conditions**

	**Current study** **(n = 109)**	**Tilford et al. 2012 [** 46 **] (n = 146)**	**Manuel et al. 2002 [** 47 **] (n = 8258)**	**Petrou et al. 2007 [** 48 **] (n = 120)**	**Smith**-**Olinde et al. 2008 [** 49 **] (n = 103)**	**Quinn et al. 2004 [** 50 **] (n = 243)**	**Boulton et al. 2006 [** 51 **] (n = 79)**
**Country study conducted in**	Hong Kong	USA	Canada	UK	USA	USA	UK
**Gender**	F: 44%	F: 15%	F:63%	F: 45%	F: 49%	F: 52%	F: 41%
**Age (Mean ± SD; Range)**	23.6 y ± 8.5 (5–53)	8.6 y ± 3.3 (4–17)	Not reported	7.9 y (5–12)	7.3 y ± 1.9 (5–10)	10 y	6.2 y ± 1.5 (3–8)
**Condition studied**	Down syndrome	Autistic spectrum disorder	Musculoskeletal	Hearing impairment	Hearing loss	Retinopathy of prematurity	vision impairment or blindness
**HUI3 score**							
**Overall**	0.55 ± 0.27	0.66 ± 0.23					
**Chronic orthopaedic**	0.47 ± 0.23 (n = 11)		0.87				
**Hearing**	0.39 ± 0.28 (n = 21)			0.63 ± 0.32	0.62 ± 0.20		
**Vision**	0.47 ± 0.29 (n = 50)					0.59 ± 0.39	0.34 ± 0.43

Further analysis relating HRQoL and chronic health conditions found that none of the physical health issues assessed suggested significant negative association with HRQoL. In contrast, hearing and behavior problems were found to significantly decrease the HRQoL of people with DS, which is actually in line with the observations of many local parents of DS children and the previous quality of life studies on subjects with hearing impairment [52,53] and with behavioral problems [22]. In spite of the existing evidence linking developmental disabilities and impaired quality of life [22], there are so far lacking evidence on its impact on DS subjects. Our findings help to bridge this knowledge gap.

Additionally, a gradient relationship was observed, with increasing number of developmental-behavioral problems resulted in more deficit of HRQoL. This is an important finding on DS subjects which were not reported previously. Developmental issues such as sensory impairments (hearing, vision) may impair individual subject’s ability to communicate, consequently provoking frustration and problem behaviors, as they are unable to express themselves. Thus, each additional developmental-behavioral problem would escalate the burden to individual subject with DS and reduce their quality of life. In addition, deteriorations in health may also be associated with an increase in aggressive behavior, particularly when there were communication difficulties in expressing underlying medical problems, as shown in previous studies on those with intellectual disabilities [54,55]. Thus, behavior problems could possibly be an indication for some undiagnosed illness, reflective by a lowered HRQoL.

Effective surveillance and appropriate interventions towards hearing impairment, behavioral problems and other relevant developmental-behavioral problems would certainly help to improve the quality of life of DS subjects. Currently, there are no health surveillance programs for adults with DS to assess their hearing or behavior problems. Health care professionals looking after subjects with DS should be alerted to look for these potential developmental-behavioral problems to minimize its adverse impact. Meanwhile, regular surveillance program and necessary therapeutic services should be implemented for people with DS.

Furthermore, the ability to quantify HRQoL has broad implications. It would be useful for healthcare professionals, social workers, and counselors to communicate to expectant parents about the realistic outcome of caring for someone with DS.

Various limiting factors warrant consideration. 1) For the study population, surveys were administered through HKDSA events, classes, and gatherings; therefore, the group of individuals with DS who are severely disabled or require intensive medical monitoring might not be included. This may result in surveying the healthier population, thus underestimating the self-reported health condition occurrence rate. 2) The survey was proxy-assessed by parents/caregivers and arguments regarding the reliability of proxy-results have been queried. However, it has been suggested that agreement and reliability tend to be valid if the survey was completed by close relatives [56], which in our case all respondents were parents or caregivers. 3) As a generic instrument, HUI was used in this study to assess the HRQoL of people with DS; concrete information on quality of life specifically related to DS may not have been included.

To the best of our knowledge, this is the first study which assessed and quantified the HRQoL of Chinese people with DS. The valuable dataset allows healthcare professionals, counselors, social workers, and caregivers to be alerted about the adverse impact of developmental-behavioral problems on the overall HRQoL of people with DS. In addition, quantifiable measure of HRQoL would also facilitate longitudinal study on well-being of DS subjects and evaluation of effectiveness of intervention programs in the near future.

## Conclusions

This study validated the HUI-Ch and was applied on people with DS in Hong Kong, which concluded with valuable information on the HRQoL and in relation to the chronic health problems incurred.

## Additional files

Additional file 1: Table S1. HUI2 Multi-Attribute Health Status Classification System. Additional file 2: Table S2. HUI3 Multi-Attribute Health Status Classification System. Additional file 3: Table S3. Description of Assigned Ranges of Overall HUI2 and HUI3 Scores to Categories of Disability: None; Mild; Moderate; and Severe.

## Abbreviations

HUI: Health Utility Index
HUI2/3: Health Utility Index Mark 2/ Mark 3
HUI-Ch: Chinese version of Health Utilities Index
DS: Down syndrome
HRQoL: Health-related Quality of Life
WHO: World Health Organization
TNO-AZL: Netherlands Organization for Applied Scientific Research Medical Centre
HUInc: Health Utilities Inc.
HKDSA: Hong Kong Down Syndrome Association
