# Supplementary material for: Validation and application of health utilities index in Chinese subjects with down syndrome
Source: Health Qual Life Outcomes. 2014 Oct 14;12:144. doi: 10.1186/s12955-014-0144-x (PMC4207901; doi:10.1186/s12955-014-0144-x)
Supplement: Additional file 1: Table S1. — HUI2 Multi-Attribute Health Status Classification System. [file 12955_2014_144_MOESM1_ESM.docx]

Additional file 1: Table S1 HUI2 Multi-Attribute Health Status Classification System

|  |  |  |
| --- | --- | --- |
| **Attribute** | **Level** | **Description** |
| SENSATION | 1 | Able to see, hear, and speak normally for age. |
|  | 2 | Requires equipment to see or hear or speak. |
|  | 3 | Sees, hears, or speaks with limitations even with equipment. |
|  | 4 | Blind, deaf, or mute. |
|  |  |  |
| MOBILITY | 1 | Able to walk, bend, lift, jump, and run normally for age. |
|  | 2 | Walks, bends, lifts, jumps, or runs with some limitations but does not require help. |
|  | 3 | Requires mechanical equipment (such as canes, crutches, braces, or wheelchair) to walk or get around independently. |
|  | 4 | Requires the help of another person to walk or get around and requires mechanical equipment as well. |
|  | 5 | Unable to control or use arms and legs. |
|  |  |  |
| EMOTION | 1 | Generally happy and free from worry. |
|  | 2 | Occasionally fretful, angry, irritable, anxious, depressed, or suffering "night terrors". |
|  | 3 | Often fretful, angry, irritable, anxious, depressed, or suffering "night terrors". |
|  | 4 | Almost always fretful, angry, irritable, anxious, depressed. |
|  | 5 | Extremely fretful, angry, irritable, anxious, or depressed usually requiring hospitalization or psychiatric institutional care. |
|  |  |  |
| COGNITIVE | 1 | Learns and remembers school work normally for age. |
|  | 2 | Learns and remembers school work more slowly than classmates as judged by parents and/or teachers. |
|  | 3 | Learns and remembers very slowly and usually requires special educational assistance. |
|  | 4 | Unable to learn and remember. |
|  |  |  |
| SELF-CARE | 1 | Eats, bathes, dresses, and uses the toilet normally for age |
|  | 2 | Eats, bathes, dresses, or uses the toilet independently with difficulty. |
|  | 3 | Requires mechanical equipment to eat, bathe, dress, or use the toilet independently. |
|  | 4 | Requires the help of another person to eat, bathe, dress, or use the toilet. |
|  |  |  |
| PAIN | 1 | Free of pain and discomfort. |
|  | 2 | Occasional pain. Discomfort relieved by non-prescription drugs or self-control activity without disruption of normal activities. |
|  | 3 | Frequent pain. Discomfort relieved by oral medicines with occasional disruption of normal activities. |
|  | 4 | Frequent pain; frequent disruption of normal activities. Discomfort requires prescription narcotics for relief. |
|  | 5 | Severe pain. Pain not relieved by drugs and constantly disrupts normal activities. |
|  |  |  |
| FERTILITY | 1 | Able to have children with a fertile spouse. |
|  | 2 | Difficulty in having children with a fertile spouse. |
|  | 3 | Unable to have children with a fertile spouse. |
|  |  |  |
| Source: | Torrance et al. Medical Care 1996, Table 1, page 706 | |
| Legend: | * - Level descriptions are worded here exactly as presented to respondents of the HUI2 preference survey. | |
| Note: | Fertility attribute is optional and not part of the standard HUI23-15Q nor HUI23-40Q questionnaire | |
