# Supplementary material for: Validation and application of health utilities index in Chinese subjects with down syndrome
Source: Health Qual Life Outcomes. 2014 Oct 14;12:144. doi: 10.1186/s12955-014-0144-x (PMC4207901; doi:10.1186/s12955-014-0144-x)
Supplement: Additional file 2: Table S2. — HUI3 Multi-Attribute Health Status Classification System. [file 12955_2014_144_MOESM2_ESM.docx]

Additional file 2: Table S2 HUI3 Multi-Attribute Health Status Classification System

|  |  |  |
| --- | --- | --- |
| **Attribute** | **Level** | **Description** |
| VISION | 1 | Able to see well enough to read ordinary newsprint and recognize a friend on the other side of the street, without glasses or contact lenses. |
|  | 2 | Able to see well enough to read ordinary newsprint and recognize a friend on the other side of the street, but with glasses. |
|  | 3 | Able to read ordinary newsprint with or without glasses but unable to recognize a friend on the other side of the street, even with glasses. |
|  | 4 | Able to recognize a friend on the other side of the street with or without glasses but unable to read ordinary newsprint, even with glasses. |
|  | 5 | Unable to read ordinary newsprint and unable to recognize a friend on the other side of the street, even with glasses. |
|  | 6 | Unable to see at all. |
|  |  |  |
| HEARING | 1 | Able to hear what is said in a group conversation with at least three other people, without a hearing aid. |
|  | 2 | Able to hear what is said in a conversation with one other person in a quiet room without a hearing aid, but requires a hearing aid to hear what is said in a group conversation with at least three other people. |
|  | 3 | Able to hear what is said in a conversation with one other person in a quiet room with a hearing aid, and able to hear what is said in a group conversation with at least three other people, with a hearing aid. |
|  | 4 | Able to hear what is said in a conversation with one other person in a quiet room, without a hearing aid, but unable to hear what is said in a group conversation with at least three other people even with a hearing aid. |
|  | 5 | Able to hear what is said in a conversation with one other person in a quiet room with a hearing aid, but unable to hear what is said in a group conversation with at least three other people even with a hearing aid. |
|  | 6 | Unable to hear at all. |
|  |  |  |
| SPEECH | 1 | Able to be understood completely when speaking with strangers or friends. |
|  | 2 | Able to be understood partially when speaking with strangers but able to be understood completely when speaking with people who know me well. |
|  | 3 | Able to be understood partially when speaking with strangers or people who know me well. |
|  | 4 | Unable to be understood when speaking with strangers but able to be understood partially by people who know me well. |
|  | 5 | Unable to be understood when speaking to other people (or unable to speak at all). |
|  |  |  |
| AMBULATION | 1 | Able to walk around the neighborhood without difficulty, and without walking equipment. |
|  | 2 | Able to walk around the neighborhood with difficulty; but does not require walking equipment or the help of another person. |
|  | 3 | Able to walk around the neighborhood with walking equipment, but without the help of another person. |
|  | 4 | Able to walk only short distances with walking equipment, and requires a wheelchair to get around the neighborhood. |
|  | 5 | Unable to walk alone, even with walking equipment. Able to walk short distances with the help of another person, and requires a wheelchair to get around the neighborhood. |
|  | 6 | Cannot walk at all. |
|  |  |  |
| DEXTERITY | 1 | Full use of two hands and ten fingers. |
|  | 2 | Limitations in the use of hands or fingers, but does not require special tools or help of another person. |
|  | 3 | Limitations in the use of hands or fingers, is independent with use of special tools (does not require the help of another person). |
|  | 4 | Limitations in the use of hands or fingers, requires the help of another person for some tasks (not independent even with use of special tools). |
|  | 5 | Limitations in use of hands or fingers, requires the help of another person for most tasks (not independent even with use of special tools). |
|  | 6 | Limitations in use of hands or fingers, requires the help of another person for all tasks (not independent even with use of special tools). |
|  |  |  |
| EMOTION | 1 | Happy and interested in life. |
|  | 2 | Somewhat happy. |
|  | 3 | Somewhat unhappy. |
|  | 4 | Very unhappy. |
|  | 5 | So unhappy that life is not worthwhile. |
|  |  |  |
| COGNITION | 1 | Able to remember most things, think clearly and solve day to day problems. |
|  | 2 | Able to remember most things, but have a little difficulty when trying to think and solve day to day problems. |
|  | 3 | Somewhat forgetful, but able to think clearly and solve day to day problems. |
|  | 4 | Somewhat forgetful, and have a little difficulty when trying to think or solve day to day problems. |
|  | 5 | Very forgetful, and have great difficulty when trying to think or solve day to day problems. |
|  | 6 | Unable to remember anything at all, and unable to think or solve day to day problems. |
|  |  |  |
| PAIN | 1 | Free of pain and discomfort. |
|  | 2 | Mild to moderate pain that prevents no activities. |
|  | 3 | Moderate pain that prevents a few activities. |
|  | 4 | Moderate to severe pain that prevents some activities. |
|  | 5 | Severe pain that prevents most activities. |
|  |  | |
| Source: | Feeny et al. *PharmacoEconomics* 1995, Table III, page 494. | |
| Legend: | * - level descriptions are worded here exactly as presented to respondents in the HUI3 preference measurement surveys (Furlong et al. 1998). | |
| Proofed and revised 2003-04-07 to match HUI and HUG websites and to match the original source documents...Torrance et al Medical Care 1996, Table 1 page 706 for HUI2 and Furlong et al Annals of Medicine 2000, Table 1 page 377 for HUI3 | | |
|  | | |
