# Supplementary material for: Validation and application of health utilities index in Chinese subjects with down syndrome
Source: Health Qual Life Outcomes. 2014 Oct 14;12:144. doi: 10.1186/s12955-014-0144-x (PMC4207901; doi:10.1186/s12955-014-0144-x)
Supplement: Additional file 3: Table S3. — Description of Assigned Ranges of Overall HUI2 and HUI3 Scores to Categories of Disability: None; Mild; Moderate; and Severe. [file 12955_2014_144_MOESM3_ESM.docx]

Additional file 3: Table S3 Description of Assigned Ranges of Overall HUI2 and HUI3 Scores to Categories of Disability: None; Mild; Moderate; and Severe

|  | Range of Overall HUI2 Scores | Range of Overall HUI3 Scores |
| --- | --- | --- |
| None | 1.00 | 1.00 |
| Mild | 0.91 though 0.99 | 0.89 though .099 |
| Moderate | 0.80 through 0.90 | 0.70 through 0.88 |
| Severe | ≤0.80 | ≤0.70 |
| Dead | 0.00 | 0.00 |

The categories of disability reflect community preferences for the following types of disability: (a) none, (b) mild, (c) moderate, and (d) severe.

1. No disability, or perfect health, occurs when all attributes (dimensions or domains) of health status are at their highest functional level (Level 1).
2. Mild disability involves situations in which at least one attribute is at a reduced level of function, a reduced level of function that can be readily corrected and / or does not prevent ay activities. For example: vision that is readily corrected by glasses or contact lenses.
3. Moderate disability involves situations in which at least one attribute is at a reduced level of function that cannot be corrected and / or prevents some activities but does not involve a profound limitation. For example: being dependent on mechanical aids for mobility. Moderate overall disability can also occur in situations in which two (2) attributes are mildly affected. For example: Level 2 HUI3 vision (sees readily with glasses) and Level 2 HUI3 hearing (hears well in quite environments without a hearing aid and in noisy environments with a hearing aid).
4. Severe disability occurs in situations in which at least one (1) attribute is at a reduced level of function that cannot be corrected and prevents many activities. Severe disability may also occur in states with four (4) attributes at reduced Level 2 function.
